# SARS-CoV-2 seroprevalence in French 9-year-old children and their parents after the first lockdown in 2020

**DOI:** 10.3389/fped.2023.1274113

**Published:** 2023-10-25

**Authors:** Marie Aline Charles, Pierre-Yves Ancel, Thierry Simeon, Laetitia Marchand-Martin, Cécile Zaros, Marie-Noelle Dufourg, Valérie Benhamou, Hélène Blanché, Jean-François Deleuze, Delphine Rahib, Nathalie Lydie, Xavier de Lamballerie, Fabrice Carrat

**Affiliations:** ^1^ UMS Elfe, Ined, Inserm, EFS, Aubervilliers, France; ^2^ Center for Research in Epidemiology and Statistics, Paris Cité University, INSERM, INRAE, Paris, France; ^3^ Fondation Jean Dausset-Centre d’Etude du Polymorphisme Humain, CEPH-Biobank, Paris, France; ^4^ Health Prevention and Promotion Department, Santé Publique France, Saint-Maurice, France; ^5^ Unité des Virus Emergents, Aix Marseille University, IRD, INSERM, IHU Méditerranée Infection, Marseille, France; ^6^ Institut Pierre-Louis d'Epidémiologie et de Santé Publique, Sorbonne Université, Inserm, Paris, France; ^7^ Département de Santé Publique, Hôpital Saint-Antoine, APHP, Paris, France

**Keywords:** COVID-19, serology, children, France, household

## Abstract

**Introduction:**

Children have been significantly less affected by COVID-19 than adults and presented with milder and less symptomatic forms of the disease. However, there has been suggestion that children older than 10 years and adolescents exhibits features closer to that of young adults. Most studies combine children in different age-groups and lack sufficient numbers to explore in detail age specificities. We report data on a population-based sample of 2,555 children at the pivotal age of 9 years.

**Methods:**

In April 2020, the participants in two French nationwide cohorts of children, Elfe and Epipage2, were invited to take part into an online survey about Covid related symptoms and family life during the lockdown. A second questionnaire was sent on May 5. This questionnaire also proposed to the child included in the cohort and to one of his/her parents to take part into a capillary blood collection for Covid serology. Families who agreed to the serological survey were sent kits for dried blood spots self-sampling (DBS) with instructions. Samples were processed with a commercial Elisa test (Euroimmun®, Lübeck, Germany) to detect anti-SARS-CoV-2 antibodies (IgG) directed against the S1 domain of the spike protein of the virus.

**Results:**

Children's acceptance rate for the serological survey was around 60%. 2,555 serological results were analyzed. The weighted prevalence of a positive Elisa Spike serology was 2.8% in 9 yr-old children (95% CI: 1.7%–4.0%). Positive serology was found in 8.6% (7.4%–9.7%) of parents who provided blood. There was a significant association (*p* < 0.001) between serology of the child and parent from the same household with an odds ratio of 13.8 (7.9–24.2).

**Discussion:**

We have shown that 9-yr old children had a lower susceptibility to SARS-Cov2 infection than adults with the initial Chinese strain, similar to younger children and estimated that around 3% of them have developed antibodies against SARS-Cov2 in France after the first wave of the Covid-19 epidemics.

## Introduction

At the beginning of the COVID-19 epidemic, initial data from China indicated that children were significantly less affected by COVID-19 than adults and more frequently presented with mild and asymptomatic forms of the disease than adults (1). This has been later confirmed by many other publications and by seroprevalence studies from different parts of the world (2, 3). However, there has been a suggestion that children older than 10 years and adolescents exhibit features closer to that of young adults (2). In the USA, the weekly incidence of COVID-19 cases among adolescents aged 12–17 years was approximately twice compared with that of children aged 5–11 years between March and September 2020 (4). Evidence demonstrated that the SARS-CoV-2 viral-specific antibody response profiles vary with age and differ between younger children, adolescents, and young adults (5).

Most studies combine children in different age groups and lack sufficient numbers to explore them in detail age specificities. Here, we report data on a population-based sample of 2,555 children at the pivotal age of 9 years to gain more insight into the transition between younger and older child-type responses to SARS-CoV-2 infection.

The data originated from the child cohorts of the SAPRIS (“SAnté, Perception, pratiques, Relations et Inégalités Sociales en population générale pendant la crise COVID-19”) project, a consortium of prospective French cohort studies involving three general population-based adult cohorts, aside from the two child cohorts (6). The goal of the present analysis is to describe (1) the symptoms compatible with a SARS-CoV-2 infection in the two involved cohorts of children born in 2011 during or right after the first lockdown in France (from 17 March 2020 to 11 May 2020) and (2) the prevalence of SARS-CoV-2 ELISA Spike seropositivity in children and one of their parents.

## Population

In April 2020, the participants in two French nationwide cohorts of children, namely, Elfe and Epipage2, were invited to take part in an online survey about COVID-19-related symptoms and family life during the lockdown.

Both cohorts had enrolled children at birth in 2011. Children born at term, moderate preterm, or late preterm (after 33 weeks of gestation) were participants in the Elfe cohort and enrolled on 25 selected days over 2011, from a random sample of 320 maternity units in mainland France (7). Children born extremely preterm (below 35 weeks) were participants in the Epipage2 cohort. All maternity units in French mainland regions (but one) and in overseas regions were involved in the Epipage2 inclusion phase over periods that lasted from 5 weeks to 8 months according to three gestational age strata (8).

All families who had not withdrawn from the cohorts and had an email address known by the management teams were eligible for the COVID-19 survey.

Elfe children born before 35 weeks of gestation were excluded from the present analyses as these preterm children are best represented by the Epipage2 cohort.

## Methods

### Data collection

The first online questionnaire was sent on 16 April 2020 and remained accessible until May 4.

For the index child, symptoms compatible with a COVID-19 infection (see list in Table 1) were reported if they had been present at least once in the last 14 days. If a symptom had been, but was no longer present when the questionnaire was completed, the duration was noted on a scale (less than 1, 1–3, 4–7, 8–14, >14 days). Finally, a question enquired about the total time (in days) between the onset of the first symptoms and the date of the questionnaire filling, but too many missing data for correct exploitation were noted. For other members of the household, only cough and/or fever in the last 14 days were reported. Additional questions included the socioeconomic and employment status of the parents, dwelling type and household composition, recurse to care for the child, preventive measures against COVID-19, familial relationship, school, and leisure-related activities of the child during the lockdown (9).

**Table 1:** Prevalence of clinical symptoms lasting at least 1 day during the lockdown according to ELISA Spike serology.

	Symptoms for at least 1 day				
	Negative or indeterminate serology		Positive serology		*p*
	*n* cases	Prevalence (%)	*n* cases	Prevalence (%)	
Fever or feverishness	37	1.5	5	7.1	<0.001
Headache	105	4.3	6	8.8	0.07
Fatigue	48	2.0	6	8.7	<0.001
Myalgia	58	2.4	5	7.1	0.01
Cough	61	2.5	3	4.5	0.31
Dyspnea	10	0.4	2	2.9	0.003
Rhinorrhea	90	3.7	1	1.5	0.34
Sore throat	55	2.2	2	2.9	0.69
Conjunctivitis and fever	2	0.1	1	1.5	0.001
Anosmia and ageusia	4	0.2	3	4.3	<0.001
Abdominal pain	140	5.7	6	9.1	0.25
Nausea and vomiting	16	0.7	0	0.0	0.50
Diarrhea	46	1.9	3	4.4	0.13
Chest pain	6	0.2	4	5.7	<0.001
Exanthema and chillains	54	2.2	2	2.9	0.69

The second questionnaire was sent on May 5 and remained available until June 29. It included the same questions as the first questionnaire and additional questions on family life and mental health. This questionnaire also proposed to the child included in the cohort and to one of his/her parents to take part in a capillary blood collection for COVID-19 serology.

### Serological tests

Families who agreed to the serological survey were sent kits with instructions for the self-sampling of dried blood spots (DBS). The cards for the parent and the child were pre-labeled. The participants added their first names and the date of sampling on the card. Before the sampling, they had to fill out an online consent and a short questionnaire about COVID-19-related symptoms in the three previous weeks and about positive PCR test(s) since the completion of the second online survey questionnaire.

After sampling, the DBS were sent in a padded pre-stamped and pre-addressed envelope to a Central Biobank (CEPH Biobank, Paris, France). On receipt, after a visual assessment of the blood spots and registration, four discs of 4.7 mm for adults and one to four discs for children were punched from the spots on a Panthera™ (PerkinElmer) and stored in 2D FluidX 96-Format 0.5 ml tubes (Brooks) at room temperature. They were sent to the virology laboratory (Unité des Virus Émergents, Marseille, France) for serological analyses.

Samples were processed with a commercial ELISA test (Euroimmun®, Lübeck, Germany) to detect anti-SARS-CoV-2 antibodies (IgG) directed against the S1 domain of the spike protein of the virus (ELISA S). The volume of eluate used corresponded to the amount of serum and dilution recommended by the manufacturer. According to the recommendation of the manufacturer, a test with an optical density ratio of ≥1.1 is ELISA-positive; if it is between 0.8 and 1.1, it is ELISA-indeterminate; and if it is <0.8, it is ELISA-negative.

Due to the low availability of serological tests at this time, kits were sent only to families living in three regions of France, from May 15 to July 6: the two regions with the highest reported cumulated rates of COVID-19, namely, Grand Est (GE) and Ile-de-France (IDF), and a region with a low reported rate, Nouvelle Aquitaine (NA) (French Public Health Agency, COVID-19 Epidemiologic Situation, 14 May 2020). From July 7, all other families who had volunteered were sent a kit. Most of the sampling were completed in the last 2 weeks of May (27%) and the first 3 weeks of July (44%), but the last kits received dated from early November.

### Ethical approval

The SAPRIS survey was approved by the Inserm Ethics Committee (approval #20–672 dated 30 March 2020). The SAPRIS-SERO study was approved by the Sud-Mediterranée III ethics committee (approval #20.04.22.74247). A specific information letter was addressed to the child in addition to the letter to the parents. Electronic consents were given by the parents for themselves and their child.

### Statistical analysis

The prevalence of SARS-CoV-2 ELISA Spike seropositivity was weighted to take into account the initial sampling plan, enrollment rate in the two cohorts, and response rate to the current survey. The weights for the two cohorts were combined to represent the target population of children born in 2011 in mainland France (Supplementary Material S1).

According to the European Centre for Disease Prevention and Control, a possible COVID-19 was defined as at least one of the following symptoms: fever, cough, dyspnea, a sudden onset of anosmia, ageusia, or dysgeusia (10). As symptoms are milder in children than those in adults, they were significantly considered as soon as they lasted for at least 1 day.

The weighted seroprevalence is presented in the total sample and according to a four-category region variable (IDF, GE, NA, and other regions).

The association between the serologies of the parent and the child was tested using the chi-square test. The odds ratio has been computed to quantify the strength of the association. The associations between the child serology and clinical symptoms were also tested using the chi-square test.

## Results

Eligible participants for the COVID-19 survey were 12,814 and 2,922 in the Elfe and Epipage2 cohorts, respectively. Among them, 3,894 (30%) and 841 (29%), respectively, responded to the second survey questionnaire and were proposed to participate in the serological survey. The acceptance rate of children for the serological survey was approximately 60% (*n* = 2,358 and 498, respectively), which was similar for the two cohorts. A total of 2,828 DBS from children were received at the Biobank. Among them, 2,680 (95%) could be punched and were sent for analysis, and 2,642 serological results were returned. After the exclusion of the missing values of the sampling date and children that could not be included in the weighing procedure, data collected from 2,555 children were included in the statistical analysis. Among 2,400, one parent had also provided a DBS that could be analyzed.

The crude and weighted characteristics of the children are presented in Table 2. The crude prevalence of a positive ELISA Spike serology was not statistically different in the preterm children of the Epipage2 cohort (2.4%) and the term children of the Elfe cohort (1.7%, *p* = 0.40); therefore, results were given for the pooled sample.

**Table 2:** Characteristics of the participant child.

	Crude		Weighted
	*n* *	%	% or mean (SD)
Cohort			
Epipage2	414	16.2	2.2
Elfe	2,141	83.8	97.8
Sex			
Female	1,261	49.4	49.2
Male	1,294	50.6	50.8
Respondent to the main questionnaire			
Mother	2,358	92.3	92.4
Other parent	196	7.6	7.6
Age of the mother in 2020, mean (SD)	2,358	40.2 (4.2)	40.0
Mother’s level of education at birth			
No university degree	510	20.2	21.2
2-year university degree	638	25.2	23.7
University degree of ≥3 years and higher	1,383	54.6	55.1
Mother’s country of birth			
France	2,404	95.0	92.4
Other European country	47	1.9	3.1
Other	79	3.1	4.5
Father’s level of education at birth			
No university degree	857	34.9	33.4
2-year university degree	538	21.9	20.9
University degree of ≥3 year and higher	1,062	43.2	45.7
Region of residence during the lockdown			
Ile-de-France	386	15.3	16.8
Grand Est	259	10.3	10.1
Nouvelle-Aquitaine	214	8.5	9.1
Other	1,660	65.9	64.0
City size			
>100,000 inhab.	326	12.9	12.9
20-000-100,000	455	18.1	18.4
<20,000 inhab.	619	24.6	25.4
Rural	1,118	44.4	43.3
Number of persons living in the household			
2 or 3	362	14.4	12.7
4 or 5	1,976	78.4	78.7
6 and +	182	7.2	8.6

* *n* with available data for the considered characteristics.

The weighted prevalence of a positive ELISA Spike serology was 2.8% in 9-year-old children (95% CI: 1.7%–4.0%). It showed the expected gradient according to the regional viral contamination rates before the lockdown, ranging from 6.9% (2.4%–11.4%) in Grand Est to 0.1% (0.0%–0.3%) in Nouvelle Aquitaine (Table 3). In the whole sample, a positive serology was found in 8.6% (7.4%–9.7%) of parents who provided blood (the mother in 76% and the father in 24% of cases), which was more than three times higher than that in their children.

**Table 3:** Crude and weighted ELISA Spike serology according to region of residence.

Children	Crude (*n*, %)						Total*	Weighted
	Negative		Indeterminate		Positive			% Positive (95% CI)
	*n*	% (95% CI)	*n*	% (95% CI)	*n*	% (95% CI)		
IDF	372	95.6 (93.6–97.7)	4	1.0 (0.0–2.0)	13	3.3 (1.6–5.1)	386	3.3 (0.6–5.9)
Grand Est	242	93.4 (90.4–96.5)	2	0.8 (0.0–1.8)	15	5.8 (2.9–8.6)	259	6.9 (2.4–11.4)
N. Aquitaine	212	98.6 (97.0–100)	2	0.9 (0–2.2)	1	0.5 (0.0–1.4)	214	0.1 (0.0–0.3)
Other regions	1,634	98.0 (97.4–98.7)	4	0.2 (0.0–0.5)	29	1.7 (1.1–2.4)	1,658	2.5 (0.9–4.0)
Total	2,460	97.2 (96.6–97.9)	12	0.5 (0.2–0.7)	58	2.3 (1.7–2.9)	2,517	2.8 (1.7–4.0)
Parents								
IDF	311	85.2 (81.6–88.9)	8	2.2 (0.7–3.7)	46	12.6 (9.2–16.0)		
Grand Est	208	86.0 (81.6–90.3)	8	3.3 (1.0–5.6)	26	10.7 (6.8–14.7)		
N. Aquitaine	183	91.5 (87.6–65.4)	3	1.5 (0.0–3.2)	14	7.0 (3.5–10.5)		
Other regions	1,398	89.4 (87.9–90.9)	49	3.1 (2.3–4.0)	117	7.5 (6.2–8.8)		
Total	2,100	88.6 (87.3–89.9)	67	2.9 (2.2–3.5)	203	8.6 (7.4–9.7)		

* Thirty-eight subjects excluded due to missing values for the region of residence.

Table 4 shows a significant association (*p* < 0.001) between the serologies of the child and the parent from the same household with an odds ratio of 13.8 (7.9–24.2).

**Table 4:** Association between ELISA Spike serologies of a 9-year-old child and a parent from the same household.

Child serology	Parent serology % (*N*)				Total	
	Positive		Negative or ind.			
	*n*	% (95% CI)	*n*	% (95% CI)	*n* ^a^	%
Positive	28	52.8 (39.3–66.4)	25	47.2 (33.6–60.7)	53	100
Negative or ind.	176	7.5 (6.4–8.6)	2,171	92.5 (91.4–93.6)	2,347	100
Total	204	8.5 (7.4–9.6)	2,196	91.5 (90.4–92.6)	2,400	100

Ind., indeterminate.

^a^ Number with serologies available for both the child and the parent.

The clinical symptoms of the “possible COVID-19” definition that lasted for at least 1 day had been more frequently declared (13.4%) during the lockdown when ELISA Spike serology was positive than when it was negative or indeterminate (3.8%, *p* = 0.02).

Among the symptoms proposed in the questionnaire, those that lasted for at least 1 day and had been significantly more frequently declared in case of positive serology were anosmia/ageusia, fever, fatigue, dyspnea, and chest pain (Table 1).

Of the children with positive serology, 74% (43/58) (CI: 63%–86%) reported no symptoms in the past 14 days (i.e., during the lockdown), and of them, 71% (CI: 58%–83%) reported no fever nor cough earlier in the year.

## Discussion

From this sample and according to ELISA Spike serology, it is estimated that 2.8% (95% CI: 1.7–4.0) of 9-year-old children born in France in 2011 had produced antibodies against the SARS-CoV-2 spike protein before the summer of 2020 in France, which is three times less than that of the parents living in the same household. In the presence of one infected parent in the household, only 14% of children had a positive serology.

In France, schools closed from 16 March 2020 to June, except for children of first-line workers. Reopening took place on 22 June 2020 for the whole country but started on May 11 in districts with low SARS-CoV-2 circulation. After the first lockdown, the incidence rate of COVID-19 as assessed by positive PCR tests in the 7 previous days decreased to below 5/100,000 inhab (11), before rising again at the end of the summer. We can assume that in our study a majority of the children and their parents with positive SARS-CoV-2 serology had been contaminated before or during the first weeks of the lockdown as most DBS were performed before the end of July.

Serological data on children after the first COVID-19 wave are scarce in France, and none has a national scope. To the best of our knowledge, only two are population-based. The first study was performed in the city north of Paris where the first COVID-19 outbreak in schools was recognized in France at the beginning of the epidemic. A serological survey performed in schools in the Haut de France region in March–April 2020 before any protection measure was taken found that 35% of 664 children aged 12–17 years who participated in the study were seropositive in high school where the outbreak occurred and only 9% of 1,340 children in primary schools of the city (12).

The second study was undertaken in the city of Nancy in a random sample of households drawn from the electoral list. It yielded a SARS-CoV-2 seropositivity prevalence of 1% (95% CI: 0.1–3.5) in 5–19-year-old children (*n* 203) in June 2020 (13). Two other studies were performed in clinical settings. In a study based on the serum samples collected from children requiring hospital admission in North Eastern France for other causes than COVID-19 between February and August 2020, seropositivity was estimated at 4.0% in 1,400 children aged ≤19 years (14). In the Ile-de-France region, 27 ambulatory pediatric practices participated in a fingerstick serological survey in April–May 2020. Data were collected from 605 children at a mean age of 5 years, of whom 204 were asymptomatic, 118 had COVID-19-compatible symptoms between 7 days and 2 months before enrollment, and 283 were symptomatic. SARS-CoV-2 seropositivity was 6.4% (95% CI: 3.4–10.7), 23.7% (16.4–32.7), and 8.5% (5.5–12.5), respectively (15). Although all these studies used different methods for SARS-CoV-2 serology, they are coherent with our regional estimates in 9-year-old children that rank from 0.1% to 6.9% in the general population depending on the intensity of SARS-CoV-2 circulation.

A number of studies have found that the seroprevalence rate is lower in children and, for some studies, in adolescents than that in adults (2). We confirmed this result in 9-year-old children and adults sharing the same household. In a household transmission survey in Geneva (16), compared with 20–49-year olds, 5–9-year olds had less than half the odds ratio of seropositivity in the presence of a positive household member, which confirms their low susceptibility to SARS-CoV-2 infections. On the other hand, in our study, parental seropositivity was seven times higher (53%) when their child was seropositive compared with negative or indeterminate. A similar finding was found in the Haut de France survey for primary school pupils in contrast to high school pupils (12). Closer contacts between young children and their parents than between adolescents and their parents has been hypothesized to explain this finding, and, in that matter, 9-year-olds seem to behave as younger children. Although we cannot determine who was the index case in the household, it was more likely the parent or another adult member of the household, given the differences in the infection rates between children and adults. This suggests that close contacts with an infected adult explained a substantial proportion of infections with the initial SARS-CoV-2 variant in 9-year-old children and therefore the high frequency of infected adults in their surroundings.

However, children have a more robust innate and/or mucosal immune response to SARS-CoV-2 than adults, which translates into the absence of adaptive immune solicitation and antibody production. The difference in seroconversion between children and adults may also be due in part to a difference in cellular immune responses (T and B cells) (17). Therefore, the absence of ELISA Spike seropositivity may not always indicate an absence of infection. In addition, our estimates are based only on ELISA Spike seropositivity, which does not have 100% specificity and sensibility, which may induce some misclassification.

The clinical symptoms associated with seropositivity in our study are in accordance with those described in other studies in children (3, 12, 18, 19) except for gastro-intestinal symptoms that were not statistically different between seropositive children and the others, although in the expected direction. Most seropositive children reported no symptoms in the first 2 weeks of the lockdown or earlier in the year. In contrast to the SAPRIS study performed in adult cohorts, only 19% of seropositive adults reported no symptoms (20), pointing again to the difference in the immune response between 9-year-old children and adults.

To conclude, we have shown that 9-year-old children had a lower susceptibility to SARS-CoV-2 infection than adults with the initial Chinese strain, similar to younger children, and estimated that approximately 3% of them have developed antibodies against SARS-CoV-2 in France after the first wave of the COVID-19 epidemics.

## Data Availability

Personal data of children from the ELFE and EPIPAGE2 cohorts cannot be made publicly available for ethical reasons. They are available upon reasonable request from the authors under data-security conditions.

## Supplementary material

The Supplementary Material for this article can be found online at: https://www.frontiersin.org/articles 10.3389/fped.2023.1274113/full#supplementary-material

## References

[1] Dong Y Mo X Hu Y Qi X Jiang F Jiang Z Epidemiology of COVID-19 among children in China. Pediatrics. (2020) 145(6):e20200702. 10.1542/peds.2020-0702 32179660

[2] Viner RM Mytton OT Bonell C Melendez-Torres GJ Ward J Hudson L Susceptibility to SARS-CoV-2 infection among children and adolescents compared with adults: a systematic review and meta-analysis. JAMA Pediatr. (2021) 175(2):143–56. 10.1001/jamapediatrics.2020.4573 32975552 PMC7519436

[3] Waterfield T Watson C Moore R Ferris K Tonry C Watt A Seroprevalence of SARS-CoV-2 antibodies in children: a prospective multicentre cohort study. Arch Dis Child. (2021) 106(7):680–6. 10.1136/archdischild-2020-320558 33172887

[4] Leeb R Price S Sliwa S Kimball A Szucs L Caruso E COVID-19 trends among school-aged children—United States, 1 March–19 September 2020. MMWR Morb Mortal Wkly Rep. (2020) 69(39):1410–5. 10.15585/mmwr.mm6939e2 33001869 PMC7537558

[5] Yang HS Costa V Racine-Brzostek SE Acker KP Yee J Chen Z Association of age with SARS-CoV-2 antibody response. JAMA Netw Open. (2021) 4(3):e214302. 10.1001/jamanetworkopen.2021.4302 33749770 PMC7985726

[6] Carrat F Touvier M Severi G Meyer L Jusot F Lapidus N Incidence and risk factors of COVID-19-like symptoms in the French general population during the lockdown period: a multi-cohort study. BMC Infect Dis. (2021) 21(1):169. 10.1186/s12879-021-05864-8 33568097 PMC7875161

[7] Charles MA Thierry X Lanoe JL Bois C Dufourg MN Popa R Cohort profile: the French national cohort of children (ELFE)—birth to 5 years. Int J Epidemiol. (2020) 49(2):368–9j. 10.1093/ije/dyz227 31747017 PMC7266552

[8] Lorthe E Benhammou V Marchand-Martin L Pierrat V Lebeaux C Durox M Cohort profile: the etude epidemiologique sur les petits ages gestationnels-2 (EPIPAGE-2) preterm birth cohort. Int J Epidemiol. (2021) 50(5):1428–9. 10.1093/ije/dyaa282 34165536 PMC8580281

[9] Pailhé A Panico L Solaz A. Children’s well-being and intra-household family relationships during the first COVID-19 lockdown in France. J Famliy Research. (2021) 34(1):249–80. 10.20377/jfr-718

[10] European Center for Diseases Control. Case definition for coronavirus disease 2019 (COVID-19), as of 29 May 2020, Available at: https://www.ecdc.europa.eu/en/covid-19/surveillance/case-definition (Accessed June 15, 2020).

[11] Santé publique France. Available at: https://geodes.santepubliquefrance.fr/#c=indicator&i=sp_ti_tp_7j.tx_pe_gliss&s=2021-10-24-2021-10-30&t=a01&view=map2. (Accessed June 15, 2020)

[12] Fontanet A Tondeur L Grant R Temmam S Madec Y Bigot T SARS-CoV-2 infection in schools in a northern French city: a retrospective serological cohort study in an area of high transmission, France, January to April 2020. Euro Surveill. (2021) 26(15):2001695. 10.2807/1560-7917 33860747 PMC8167414

[13] Gegout Petit A Jeulin H Legrand K Jay N Bochnakian A Vallois P Seroprevalence of SARS-CoV-2, symptom profiles and sero-neutralization in a suburban area, France. Viruses. (2021) 13(6):1076. 10.3390/v13061076 34200070 PMC8230202

[14] Woudenberg T Pelleau S Anna F Attia M Donnadieu F Gravet A Humoral immunity to SARS-CoV-2 and seasonal coronaviruses in children and adults in north-eastern France. EBioMedicine. (2021) 70:103495. 10.1016/j.ebiom.2021.103495 34304047 PMC8299153

[15] Cohen R Jung C Ouldali N Sellam A Batard C Cahn-Sellem F Assessment of SARS-CoV-2 infection by reverse transcription-PCR and serology in the Paris area: a cross-sectional study. BMJ Paediatr Open. (2020) 4(1):e000887. 10.1136/bmjpo-2020-000887 33665371 PMC7778737

[16] Bi Q Lessler J Eckerle I Lauer SA Kaiser L Vuilleumier N Insights into household transmission of SARS-CoV-2 from a population-based serological survey. Nat Commun. (2021) 12(1):3643. 10.1038/s41467-021-23733-5 34131124 PMC8206123

[17] Toh ZQ Anderson J Mazarakis N Neeland M Higgins RA Rautenbacher K Comparison of seroconversion in children and adults with mild COVID-19. JAMA Netw Open. (2022) 5(3):e221313. 10.1001/jamanetworkopen.2022.1313 35262717 PMC8908077

[18] Tonshoff B Muller B Elling R Renk H Meissner P Hengel H Prevalence of SARS-CoV-2 infection in children and their parents in southwest Germany. JAMA Pediatr. (2021) 175(6):586–93. 10.1001/jamapediatrics.2021.0001 33480966 PMC7823424

[19] Blankenberger J Haile SR Puhan MA Berger C Radtke T Kriemler S Prediction of past SARS-CoV-2 infections: a prospective cohort study among Swiss schoolchildren. Front Pediatr. (2021) 9:710785. 10.3389/fped.2021.710785 34485200 PMC8415623

[20] Carrat F de Lamballerie X Rahib D Blanche H Lapidus N Artaud F Antibody status and cumulative incidence of SARS-CoV-2 infection among adults in three regions of France following the first lockdown and associated risk factors: a multicohort study. Int J Epidemiol. (2021) 50(5):1458–72. 10.1093/ije/dyab110 34293141 PMC8344948

