# Supplementary material for: SARS-CoV-2 seroprevalence in French 9-year-old children and their parents after the first lockdown in 2020
Source: Front Pediatr. 2023 Oct 25;11:1274113. doi: 10.3389/fped.2023.1274113 (PMC10634499; doi:10.3389/fped.2023.1274113)
Supplement: Supplementary file 1 [file Datasheet1.pdf]

Supplementary material

Weighting of the Elfe and Epipage2 samples

Elfe sample

A statistical weight was calculated for each child for the maternity survey according to the inclusion procedure and the initial non-response, called maternity weight. The maternity weight for each infant were obtained in three steps (**equation 1**). First, each maternity unit was weighted (P1) by the inverse of probability of inclusion according to their size (defined by strata), then by an adjustment coefficient for the non-participation of maternity units using variables common to participants and non-participant units (maternity unit size, area, level of medical care, legal status). Second, each inclusion period was weighted (P2) according to maternity unit size (defined by strata), inclusion periods and number of days of inclusion periods. Third, each participating newborn was weighted (P3) according to an adjustment coefficient for mother's non-response using variables common to respondents and non-respondents (mother's year of birth, gestational age, area of residence, socio-professional category, mother's employment during pregnancy, twinship and primiparity). Then an under-coverage coefficient, which takes into account that some eligible mothers were not contacted (number of eligible infants/number of infants included in the survey), was applied.

$Weight_{maternity} = P1 \times P2 \times P3$ (_1_)

For our study population, the fitted weight for each child was calculated by multiplying the maternity weight for each child with an adjustment coefficient for the non-response of the child to the SAPRIS serology survey (**equation 2**). This adjustment coefficient was calculated from estimated probabilities of response obtained by logistic regression including variables common to respondents and non-respondents (birth preparation sessions, father's employment at birth, father's age, mother's marital status at birth, alcohol consumption during pregnancy, twinship, mother's employment at birth). These probabilities were ordered to obtain sorted scores used to constitute homogeneous response groups in which the non-response was considered to be random. Thus, the maternity weight of respondents with a low probability of responding and uncommon characteristics was increased.

$Weight_{study\_pop} = Weight_{maternity} \times adjustment\_coefficient\_for\_non{-}response$ (2)

Finally, a calibration on margins was performed on the study population weights by using auxiliary variables (mother's age, region, life in couple, migration status, level of education and primiparity) obtained from the 2011 state register's statistical data and the 2010 French National Perinatal study and four variables from the closest available population at the time of the survey (2016) (type of dwelling, urban/rural city, region of current residence, type of household) for households with a child born in 2011.

Because of a specific attrition due to the low rate of respondents in our population study, the weights considered as extreme were truncated, which induced a bias but decreased the variance and the range of weights (22).

The weights reconstitute the population of children born after 33 weeks of gestation in metropolitan France in 2011

Epipage2 sample

A statistical weight was calculated for each child surviving after initial neonatal hospitalisation according to the inclusion procedure. A P1 weight for each infant corrected for the sampling procedure (different duration of inclusion periods in different gestational age (GA) strata) by attribution of the following values 1, 35/26, 35/5 for respectively the 22-26 GA, 27-31 GA and 23-24GA strata. As the acceptation rate was high for the Epipage2 cohort, no correction for initial participation rate in the cohort was undertaken. Second a fitted weight for each child was calculated by multiplying the P1 weight for each child with a P2 adjustment coefficient for the response of the child to the SAPRIS serology survey (**equation 2**). This adjustment coefficient was calculated from estimated inverse probabilities of response obtained by logistic regression including the following variables common to respondents and non-respondents (region of birth, gestational age strata, multiple pregnancy, maternal age strata, maternal country of birth (France yes or no), socio-professional class, partner at birth, educational level strata, primiparity, child sex, severe neonatal morbidity). Finally, a calibration on margins was performed on the study population weights by using the same variables used for P2.

The weights reconstitute the population of children born between 26 and 34 weeks of gestation weeks of gestation in France in 2011.

Total sample

The 37 children born between 33-34 weeks of gestation were excluded from the Elfe sample to obtain a sample disjoint from the Epipage 2 sample. The total population is simply estimated at the sum of the two samples
